# Perioperative outcomes of robot-assisted, laparoscopic or thoracoscopic, and open surgery across 13 cancer operations: a single-institution cohort of 36,208 cases

**DOI:** 10.1007/s11701-026-03814-7

**Published:** 2026-08-17

**Authors:** Hojun Yu, Kyung Hee Han, Soyeoun Kim, Chang Wook Jeong

**Affiliations:** 1 Office of Hospital Information, Seoul National University Hospital, Seoul, Republic of Korea; 2 Interdisciplinary Program in Medical Informatics, Seoul National University, Seoul, Republic of Korea; 3 Department of Obstetrics and Gynecology, Seoul National University Hospital, Seoul, Republic of Korea; 4 Seoul National University Hospital Biomedical Research Institute, Seoul, Republic of Korea; 5 Department of Urology, Seoul National University Hospital, Seoul, Republic of Korea

**Keywords:** Robotic surgical procedures, Minimally invasive surgical procedures, Surgical oncology, Propensity score, Comparative effectiveness research, Postoperative complications

## Abstract

To compare perioperative outcomes of robot-assisted surgery, video-assisted (laparoscopic or thoracoscopic) surgery and open surgery across 13 oncologic procedure groups, because aggregate technology-level comparison is confounded by procedure mix and by the calendar time at which each specialty adopted robotics. In this single-institution retrospective cohort study, 36,208 index oncologic procedures in 34,785 patients (January 2016 to December 2025) were identified from the institutional clinical data warehouse. The whole cohort and 22 procedure-level pairwise comparisons were adjusted by stabilized inverse probability of treatment weighting, with indication-specific cancer-site adjustment where tumor stage was not comparable across arms. The primary outcome was major complications (Clavien–Dindo grade ≥ 3); secondary outcomes were perioperative transfusion, length of stay, 30-day readmission and conversion to open surgery. The robot-assisted share rose from 16.8% in 2016 to 48.2% in 2025, while the open share fell to 19.4%. After whole-cohort weighting, all covariates reached a standardized mean difference of 0.10 or less. Major complications were lower for robot-assisted than open partial nephrectomy (odds ratio 0.33, 95% confidence interval 0.19 to 0.55) and radical prostatectomy (0.24, 0.13 to 0.42). Transfusion was lower in 14 of 22 comparisons and length of stay in 13, the largest reduction being robot-assisted radical cystectomy (geometric mean ratio 0.64). Robot-assisted and video-assisted approaches were associated with fewer complications and transfusions and shorter stay than open surgery, most robustly for partial nephrectomy and radical prostatectomy. Procedure-level rather than aggregate evidence should guide interpretation and robotic investment.

**Supplementary Information:**

The online version contains supplementary material available at 10.1007/s11701-026-03814-7.

## Introduction

The use of robot-assisted surgery (RAS) for oncologic procedures continues to rise [1–4]. However, high-quality evidence comparing it directly with open and laparoscopic surgical approaches is often lacking. For example, recent systematic reviews and meta-analyzes have compared all three approaches for thoracic surgery [5–9]; similar meta-analyzes are lacking for other surgical categories. Prospective trials and registries of robotic oncologic surgery offer high-fidelity data but are typically confined to a single specialty or procedure. Studying procedures in isolation precludes any characterization of the relative perioperative benefits of RAS across an institution’s full oncologic portfolio.

A comprehensive evaluation can help inform institutional investment in RAS for oncology. A direct comparison of RAS versus video-assisted surgery (VAS, comprising laparoscopic and thoracoscopic approaches) versus open surgery would be revealing. However, a naive whole-hospital comparison of the three approaches would be misleading, because procedure mix and the timing of RAS adoption across oncologic disciplines confound the result.

This paper offers a complementary approach. We conducted a ten-year retrospective cohort study at Seoul National University Hospital (SNUH), a high-volume tertiary academic center, to characterize the use and perioperative benefits of all three surgical approaches across 13 oncologic procedure groups. In doing so, we sought to answer one question: after accounting for procedure mix and calendar-time adoption, does RAS confer procedure-specific perioperative benefits?

By creating a common analytic framework from a single clinical data warehouse (CDW), we frame the comparison at the procedure level, so that the resulting evidence can inform institution-level decisions about where investment in RAS yields the greatest perioperative return.

## Methods

### Study setting

This retrospective cohort study used the CDW of SNUH, a tertiary academic center with active oncologic RAS programs across urology, general surgery, thoracic surgery, gynecologic oncology and head-and-neck surgery. The study was approved by the institutional review board (IRB-E-2602-021-1714) and registered with the institutional Data Review Board (DRB-E(I)-2026-01-02). Informed consent was waived for retrospective analysis. Reporting follows the STROBE statement [10].

### Study design

**Fig. 1:**
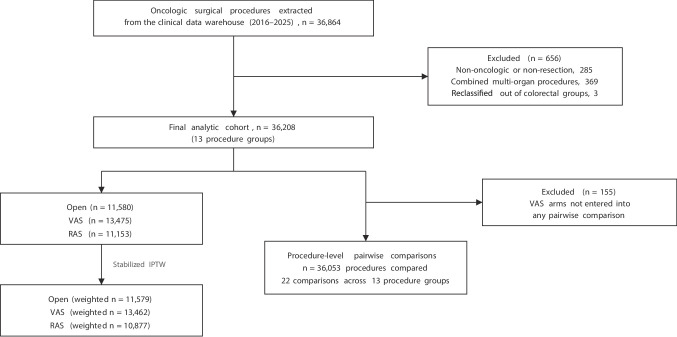
Study flow diagram. Stabilized inverse probability of treatment weighting was applied to the whole cohort for the baseline summary; for the procedure-level pairwise comparisons, weights were estimated separately within each procedure group. One procedure met both the clinical-grounds and combined-procedure exclusion criteria and is counted once in the total. Weighted group totals are those used for Table 1. CDW clinical data warehouse, IPTW inverse probability of treatment weighting, RAS robot-assisted surgery, VAS video-assisted surgery

All patients who underwent index oncologic surgery between January 2016 and December 2025 were identified from the CDW. Within the active oncologic RAS programs, 13 procedure groups were specified a priori: radical prostatectomy, gastrectomy, colectomy, low anterior resection or total mesorectal excision (LAR/TME), lung lobectomy (including segmentectomy), hysterectomy (including total abdominal hysterectomy with bilateral salpingo-oophorectomy and combined procedures), partial nephrectomy, radical nephrectomy, radical cystectomy, nephroureterectomy, thyroidectomy, pylorus-preserving pancreatoduodenectomy (PPPD) and esophagectomy. The cohort was assembled from operative scheduling records and cross-referenced against Health Insurance Review and Assessment Service (HIRA) billing codes. From 36,864 procedures initially identified, 656 were excluded — 285 on clinical grounds (non-oncologic or non-resectional cases and misclassified primary site), 369 combined multi-organ procedures and 3 robotic colorectal cases reclassified as non-colorectal — yielding a final analytic cohort of 36,208 index oncologic procedures (Fig. 1). One procedure met both the clinical-grounds and the combined-procedure criteria and is counted once.

**Table 1:** Baseline characteristics and perioperative outcomes by surgical approach after adjustment

Variable	Open (*n* = 11,580)	VAS (*n* = 13,475)	RAS (*n* = 11,153)	SMD (weighted)ᵃ	*P* valueᵇ
*Baseline characteristicsᶜ*					
Age, years (mean ± SD)	60.7 ± 13.2	61.0 ± 12.1	60.4 ± 13.5	0.045	0.002
Body mass index, kg/m²	24.2 ± 3.3	24.2 ± 3.2	24.2 ± 3.2	0.015	0.460
Charlson comorbidity index	2.2 ± 0.7	2.2 ± 0.6	2.2 ± 0.8	0.003	0.965
Pathologic T stage (numeric)ᵈ	1.9 ± 1.0	1.9 ± 1.0	1.9 ± 0.8	0.071	< 0.001
Female sex	5,349 (46.2)	6,107 (45.4)	4,772 (43.8)	0.048	0.002
ASA physical status					< 0.001
1–2	10,233 (88.4)	12,042 (89.4)	9,817 (90.2)		
≥ 3	1,346 (11.6)	1,420 (10.6)	1,060 (9.8)	0.061	
Prior abdominal or thoracic surgery	154 (1.3)	174 (1.3)	150 (1.4)	0.008	0.837
Prior cancer surgery	468 (4.0)	552 (4.1)	434 (4.0)	0.006	0.910
Calendar year of surgeryᵉ					0.384
Early (2016–2020), weighted *n* = 17,350	5,687 (32.8)	6,521 (37.6)	5,142 (29.6)	0.017	
Mid (2021–2023), weighted *n* = 12,346	3,807 (30.8)	4,692 (38.0)	3,846 (31.1)		
Late (2024–2025), weighted *n* = 6,223	2,084 (33.5)	2,249 (36.2)	1,889 (30.3)		
*Adjusted perioperative outcomes*					
Major complication (CDC ≥ 3), n (%)	564 (4.87)	468 (3.47)	370 (3.41)	–	< 0.001
Perioperative transfusion, n (%)	2,053 (17.71)	874 (6.48)	933 (8.58)	–	< 0.001
30-day readmission, n (%)	324 (2.80)	305 (2.26)	239 (2.20)	–	0.005
In-hospital mortality (CDC 5), n (%)	34 (0.29)	15 (0.11)	10 (0.09)	–	< 0.001
Length of stay, days (weighted median)	6	7	5	–	< 0.001
Estimated blood loss, mL (weighted median)	210	100	155	–	< 0.001
Operation time, min (weighted median)	124	153	140	–	< 0.001
Conversion to open or thoracotomy, n (%)	–	124 (0.92)	19 (0.17)	–	< 0.001

ᵃBalance was assessed by the standardized mean difference (SMD); a weighted SMD of 0.10 or less indicates adequate balance. Weighted P values are reported for completeness; in a cohort of this size, detected differences are negligible by the SMD criterion and should not be read as meaningful imbalance

ᵇP values are from weighted linear (continuous) or logistic (categorical) models comparing the three approaches

ᶜValues are stabilized inverse probability of treatment weighting (IPTW)-weighted means ± SD for continuous variables, or weighted medians for skewed measures as labeled, and weighted n (%) for categorical variables (weighted group totals: open 11,579, VAS 13,462, RAS 10,877), estimated from a single treatment-probability model fitted to the whole cohort

ᵈPathologic T stage is reported for the subset with comparable TNM staging (*n* = 31,306). Procedures staged by other systems, such as hysterectomy, are excluded from this row

ᵉCalendar year is categorical; each period row shows the within-period approach composition, so rows sum to 100%. The SMD is computed on year as a continuous covariate

ASA American Society of Anesthesiologists, CDC Clavien–Dindo classification, RAS robot-assisted surgery, SMD standardized mean difference, VAS video-assisted surgery

### Data sources and collection

All data were extracted from SUPREME 2.0, the CDW of SNUH, a platform that uses real-time machine learning and natural language processing to support clinical studies. Change-data-capture and extract-transform-load pipelines collect, distribute and synchronize data across departments and platforms in near real time. Linked data are organized into 12 clinical domains and 25 subject areas conforming to the HL7 FHIR standard. As of January 2026, SUPREME 2.0 held records for approximately 6.9 million patients.

The present analysis used a fixed data extract of procedures performed through December 2025. Procedures and surgical approach were identified from operative records and cross-referenced against HIRA billing codes. ICD-10 codes provided anatomical information on cancer site. Admission and discharge records provided patient demographics including body mass index (BMI), comorbidities, American Society of Anesthesiologists (ASA) physical status, prior surgical history, length of stay (LOS) and 30-day readmission. Anesthesia records provided operation times and estimated blood loss. Transfusion records confirmed perioperative allogeneic transfusion. Pathology records were the primary source for pathologic T stage, supplemented for urologic cases by the institutional prospective urology registry. Emergency department and intensive care unit records documented severe complications.

### Classification of surgical approaches

HIRA billing codes were the authoritative source for surgical approach and were cross-referenced against operative records. Approaches were classified as open, VAS or RAS. The da Vinci platform was used for all RAS procedures.

### Outcomes

The primary endpoint was major complications, defined as Clavien–Dindo (CDC) grade ≥ 3 events during the index hospitalization. Secondary endpoints were perioperative allogeneic transfusion, LOS, 30-day all-cause readmission, conversion to an open approach and in-hospital mortality (CDC grade 5). Estimated blood loss and operation time were recorded but, being post-treatment variables, were reported descriptively only.

Complications were graded with the CDC classification [11]. Grade ≥ 3 represents the composite of grades 3–5; in-hospital deaths are therefore contained within that composite and are additionally reported separately as in-hospital mortality, so the two outcomes are not mutually exclusive.

### Statistical analysis

Continuous variables are summarized as mean (SD), or as median (IQR) for right-skewed measures (LOS, estimated blood loss and operation time); categorical variables are summarized as weighted number (%). Covariate balance was quantified by the standardized mean difference (SMD) before and after weighting. Binary outcomes were analyzed on the odds-ratio scale and right-skewed continuous outcomes on the geometric-mean-ratio scale.

Surgical approach is confounded by procedure mix and calendar time. We addressed this at two levels. For the whole-cohort summary (Table 1), a single stabilized inverse probability of treatment weighting (IPTW) model was fitted to all 36,208 procedures, estimating each patient’s probability of the received approach from the covariates below and reweighting the cohort to remove baseline imbalance.

For each of the 22 procedure-level pairwise comparisons, a separate stabilized-IPTW model was fitted within the corresponding procedure group. Base covariates were age, sex, Charlson Comorbidity Index [12], BMI, ASA physical status, prior surgical history, prior oncologic surgery within the cohort and calendar year (linear and quadratic terms). Pathologic T stage was included in groups with at least 50% ascertainment. Four groups received indication-specific cancer-site flags in lieu of T stage, because tumor stage was not comparable across arms for nephroureterectomy (bladder C67; renal pelvis C65), pancreatoduodenectomy (pancreas C25), colectomy (right colon C18.0–C18.4; left colon C18.5–C18.7) and hysterectomy (cervical C53; endometrial C54–C55; ovarian C56).

Stabilized weights were computed as the marginal probability of the surgical approach divided by its conditional probability given the covariates, with conditional probabilities trimmed at the 1st and 99th percentiles to limit extreme weights [13]. Balance was assessed by the maximum absolute SMD across approaches.

Comparisons were classified by the maximum SMD as adequate (≤ 0.10), marginal (0.10–0.20) or exploratory (> 0.20) [13–16]. Balance was judged by the SMD rather than by the P value, because the SMD is independent of sample size; weighted P values are reported in the tables for completeness.

Binary outcomes were modeled by weighted logistic regression with HC3 robust standard errors; LOS and operation time were modeled by weighted log-linear regression, yielding geometric mean ratios. Multiple imputation addressed missing covariates (BMI, estimated blood loss, prior non-oncologic surgical history and pathologic T stage). Chained equations with predictive mean matching generated ten datasets; models were fitted within each and estimates pooled by Rubin’s rules. To avoid overfitting for RAS versus open esophagectomy (*n* = 238 versus *n* = 39), that comparison was refitted with median imputation and L2-penalized propensity estimation. All analyzes were performed in Python (version 3.11) using the pandas, NumPy, SciPy, scikit-learn and statsmodels packages. A large language model (Claude Opus 4.8; Anthropic) was used for English-language editing and for formatting and assembling tables and figures.

## Results

### Whole-cohort overview

The analytic cohort comprised 36,208 index oncologic procedures: open in 11,580 (32.0%), VAS in 13,475 (37.2%) and RAS in 11,153 (30.8%) (Table 1). Mean (SD) age was 61.0 (12.7) years and 45.4% of patients were female. Baseline age, sex and T stage differed across approach groups (Table 1; unadjusted detail in Online Resource 1), reflecting heterogeneous procedure-group composition and temporal confounding rather than simple between-approach differences. After procedure-stratified stabilized weighting, within-procedure balance improved substantially, with all measured covariates balanced to an SMD of 0.10 or less (Table 1; Online Resource 2), even though procedure mix remains structurally tied to approach. This whole-cohort view is for descriptive context only: surgical approach is confounded by both procedure and era, so a single aggregate comparison would be misleading.

### Procedure-level distribution and outcomes

Surgical approach distribution and unadjusted perioperative outcomes for each of the 13 procedure groups are shown in Online Resource 3. RAS predominance was greatest in radical prostatectomy and thyroidectomy, whereas open surgery remained common in radical cystectomy, esophagectomy and pancreatoduodenectomy. These patterns indicate that approach availability and selection differ markedly by procedure, reinforcing the need for within-procedure comparison.

### Procedure-level stabilized IPTW pairwise comparisons

Twenty-two pairwise comparisons were analyzed by stabilized IPTW (Table 2, the analytic unit of this study; forest plots in Fig. 3a, b). Adequate balance (maximum SMD ≤ 0.10) was achieved in 7 comparisons: radical nephrectomy VAS versus open, nephroureterectomy VAS versus open, gastrectomy VAS versus open, PPPD RAS versus open, colectomy VAS versus open, LAR/TME VAS versus open and hysterectomy VAS versus open. Marginal balance was achieved in 6 comparisons, including partial nephrectomy RAS versus open and radical cystectomy RAS versus open. Nine comparisons yielded exploratory balance, in which residual imbalance was dominated by calendar year, reflecting robotic adoption. Covariate balance before and after weighting is summarized in Online Resource 2.

**Table 2:** Procedure-level pairwise comparisons adjusted by stabilized inverse probability of treatment weighting

Procedure	Comparison	*n* (treated / reference)	maxSMD	Balanceᵃ	CDC ≥ 3 OR (95% CI)	Transfusion OR (95% CI)	LOS GMR (95% CI)	30-day readmission OR (95% CI)	Conversion to open OR (95% CI)ᵇ	Operation time GMR (95% CI)ᶜ
*Urology*										
Radical prostatectomy	RAS vs. open	4,386 / 464	0.313	✗	0.24 (0.13–0.42)	0.09 (0.07–0.11)	0.95 (0.93–0.98)	0.31 (0.15–0.64)	NA	0.97 (0.94–1.00)
Partial nephrectomy	RAS vs. open	1,560 / 755	0.109	△	0.33 (0.19–0.55)	0.50 (0.38–0.65)	0.93 (0.91–0.95)	0.30 (0.17–0.54)	NA	0.91 (0.88–0.95)
Partial nephrectomy	RAS vs. VAS	1,560 / 155	0.129	△	1.74 (0.25–12.06)	1.32 (0.63–2.77)	0.99 (0.96–1.02)	2.21 (0.19–25.00)	RAS 0/1,560; VAS 1/155	1.03 (0.97–1.10)
Radical nephrectomy	RAS vs. open	211 / 564	0.457	✗	0.23 (0.06–0.85)	0.25 (0.16–0.39)	0.76 (0.72–0.79)	0.44 (0.09–2.24)	NA	0.81 (0.76–0.86)
Radical nephrectomy	VAS vs. open	369 / 564	0.019	✓	0.51 (0.25–1.03)	0.24 (0.17–0.34)	0.84 (0.81–0.88)	0.46 (0.16–1.35)	NA	0.84 (0.81–0.88)
Radical cystectomy	RAS vs. open	675 / 404	0.102	△	0.99 (0.70–1.39)	0.34 (0.26–0.43)	0.64 (0.57–0.71)	0.74 (0.43–1.29)	NA	1.07 (1.01–1.13)
Nephroureterectomy	RAS vs. open	75 / 258	0.715	✗	2.00 (0.75–5.37)	0.44 (0.18–1.05)	1.07 (0.86–1.32)	2.06 (0.66–6.49)	NA	1.08 (0.95–1.22)
Nephroureterectomy	VAS vs. open	104 / 258	0.050	✓	0.45 (0.13–1.60)	0.58 (0.32–1.05)	0.91 (0.85–0.97)	0.37 (0.07–2.06)	NA	1.07 (1.00–1.15)
*General surgery —upper gastrointestinal and hepatobiliary*										
Gastrectomy	RAS vs. open	1,043 / 1,252	0.140	△	0.68 (0.45–1.03)	0.20 (0.13–0.31)	0.89 (0.86–0.92)	0.60 (0.34–1.05)	NA	1.16 (1.13–1.19)
Gastrectomy	VAS vs. open	4,133 / 1,252	0.019	✓	0.67 (0.49–0.90)	0.35 (0.28–0.44)	0.89 (0.86–0.91)	0.76 (0.52–1.13)	NA	0.98 (0.96–1.00)
Pancreatoduodenectomy	RAS vs. open	887 / 1,365	0.094	✓	1.38 (1.08–1.78)	0.48 (0.39–0.58)	0.81 (0.78–0.84)	1.63 (1.20–2.22)	NA	1.02 (0.99–1.05)
*General surgery—colorectal*										
Colectomy	RAS vs. open	31 / 1,382	0.810	✗	0.46 (0.06–3.72)	1.23 (0.43–3.54)	0.99 (0.88–1.11)	0.49 (0.03–7.27)	NA	2.13 (1.87–2.43)
Colectomy	VAS vs. open	1,263 / 1,382	0.023	✓	0.75 (0.56–1.01)	0.74 (0.59–0.92)	1.02 (0.99–1.05)	0.97 (0.68–1.39)	NA	1.26 (1.22–1.29)
Low anterior resection / TME	RAS vs. open	188 / 1,171	0.394	✗	1.34 (0.74–2.41)	0.86 (0.50–1.49)	1.05 (1.00–1.10)	1.91 (1.00–3.64)	NA	2.19 (2.08–2.32)
Low anterior resection / TME	VAS vs. open	932 / 1,171	0.021	✓	0.98 (0.70–1.38)	0.66 (0.48–0.90)	1.14 (1.11–1.17)	1.00 (0.65–1.53)	NA	1.38 (1.33–1.42)
Low anterior resection / TME	RAS vs. VAS	188 / 932	0.251	✗	1.39 (0.77–2.52)	1.51 (0.84–2.74)	0.93 (0.89–0.97)	1.91 (0.99–3.71)	RAS 0/188; VAS 6/932	1.60 (1.53–1.68)
*Thoracic surgery*										
Lung lobectomy	RAS vs. open	336 / 251	0.488	✗	0.91 (0.31–2.67)	0.03 (0.02–0.07)	0.79 (0.67–0.92)	1.52 (0.34–6.82)	NA	0.84 (0.74–0.95)
Lung lobectomy	RAS vs. VAS	336 / 3,080	0.155	△	0.86 (0.40–1.84)	1.44 (0.71–2.94)	0.99 (0.93–1.05)	1.03 (0.42–2.52)	0.76 (0.23–2.49)	1.33 (1.27–1.38)
Esophagectomy	RAS vs. open	238 / 39	0.369	✗	1.33 (0.19–9.45)	0.38 (0.17–0.83)	0.85 (0.64–1.14)	0.69 (0.22–2.21)	NA	1.48 (0.91–2.41)
*Gynecology*										
Hysterectomy	RAS vs. open	330 / 842	0.326	✗	0.48 (0.22–1.03)	0.32 (0.23–0.46)	0.79 (0.73–0.86)	0.93 (0.36–2.36)	NA	1.07 (0.98–1.17)
Hysterectomy	VAS vs. open	3,284 / 842	0.040	✓	0.71 (0.42–1.18)	0.34 (0.28–0.41)	0.73 (0.71–0.74)	1.11 (0.55–2.23)	NA	0.93 (0.90–0.95)
*Endocrine surgery*										
Thyroidectomy	RAS vs. open	1,193 / 2,833	0.151	△	1.87 (0.56–6.27)	0.69 (0.23–2.13)	1.03 (1.02–1.05)	3.68 (0.87–15.48)	NA	1.70 (1.67–1.74)

ᵃBalance: adequate (maxSMD ≤ 0.10), marginal (0.10 < maxSMD ≤ 0.20), exploratory (maxSMD > 0.20). ✓ adequate, △ marginal, ✗ exploratory

ᵇConversion to open is reported as an odds ratio only for RAS-versus-VAS comparisons and is not applicable (NA) to RAS-versus-open and VAS-versus-open comparisons. Where the robotic arm had zero conversions, raw counts are shown

ᶜOperation time (skin-to-skin, from anesthesia records) is a post-treatment mediator, reported as a descriptive secondary outcome only; causal interpretation is not appropriate

All estimates are from stabilized inverse probability of treatment weighting models and are reported as odds ratios (OR) or geometric mean ratios (GMR) with 95% confidence intervals (CI). Robotic colorectal cases (colectomy and low anterior resection / TME) were reclassified by ICD-10 primary diagnosis because the institutional billing code for robotic colorectal resection does not distinguish colon from rectum. Statistical significance corresponds to a 95% CI that excludes 1.0; because balance was judged by the standardized mean difference rather than by P values, exact P values are not tabulated

CDC Clavien–Dindo classification, CI confidence interval, GMR geometric mean ratio, LOS length of stay, maxSMD maximum standardized mean difference, OR odds ratio, RAS robot-assisted surgery, TME total mesorectal excision, VAS video-assisted surgery

In most comparisons, perioperative transfusion was reduced with RAS, most markedly for RAS versus open radical prostatectomy (odds ratio 0.09, 95% confidence interval 0.07–0.11), gastrectomy (0.20, 0.13–0.31) and radical cystectomy (0.34, 0.26–0.43). RAS versus open partial nephrectomy, at marginal balance, showed concurrent reductions in major complications (0.33, 0.19–0.55), transfusion (0.50, 0.38–0.65) and 30-day readmission (0.30, 0.17–0.54), with shorter LOS (geometric mean ratio 0.93, 0.91–0.95) — the most comprehensive multi-domain benefit observed.

Nephroureterectomy VAS versus open, the best-balanced urologic contrast (maximum SMD 0.050), showed shorter LOS (geometric mean ratio 0.91, 0.85–0.97) without a significant difference in major complications (0.45, 0.13–1.60) or transfusion (0.58, 0.32–1.05). RAS versus open radical cystectomy showed the largest reduction in LOS (geometric mean ratio 0.64, 0.57–0.71) without a significant complication difference (odds ratio 0.99). RAS versus open radical prostatectomy, although exploratory on balance owing to calendar-year confounding, showed consistent reductions across complications (0.24), transfusion (0.09) and readmission (0.31).

In contrast, RAS versus open pancreatoduodenectomy — despite adequate balance (maximum SMD 0.094) — showed higher major complications (odds ratio 1.38, 1.08–1.78) and 30-day readmission (1.63, 1.20–2.22) alongside reduced transfusion (0.48) and shorter LOS (geometric mean ratio 0.81). A prespecified period-stratified analysis (Online Resource 6) localized the elevated complication and readmission signals to the 2021–2023 program-expansion period, with resolution toward the null in 2024–2025. This is consistent with a learning-curve or transient case-selection effect rather than an intrinsic disadvantage of the robotic approach.

### Temporal adoption trajectories

**Fig. 2:**
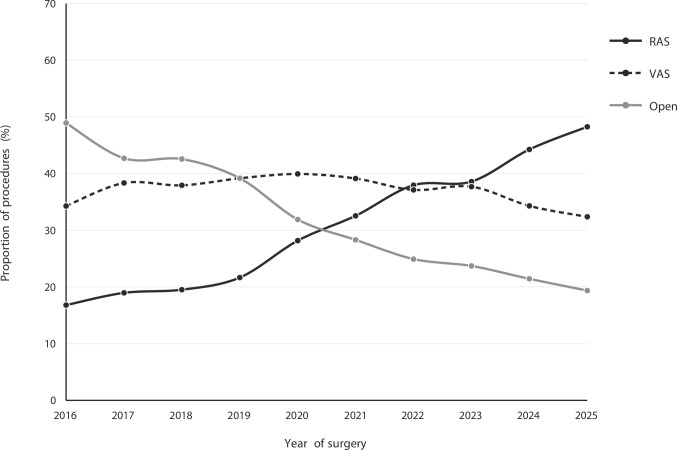
Surgical approach composition by year (2016–2025) across the whole cohort, shown as within-year proportions. RAS robot-assisted surgery, VAS video-assisted surgery

Across the whole cohort, the RAS share rose from 16.8% in 2016 to 48.2% in 2025, while the open share fell from 48.9% to 19.4%. The VAS share remained relatively stable (Fig. 2). Procedure-level trajectories (Online Resource 4) revealed markedly different maturation. RAS adoption was early and near-complete in radical prostatectomy (by 2020) and more recent in partial nephrectomy (by the mid-2020s), but nascent in radical nephrectomy, lung lobectomy and esophagectomy. These divergent trajectories are the structural source of the residual calendar-year imbalance seen in the exploratory comparisons and explain why procedure-level, time-aware adjustment is essential (.

## Discussion

This single-institution study of 36,208 oncologic procedures, structured as a funnel from a whole-cohort overview to procedure-level stabilized IPTW comparisons, indicates that surgical approach cannot be appraised as a single technology. Approach is structurally confounded by procedure mix and by calendar time, as the whole-cohort summary (Table 1) and the divergent adoption trajectories (Fig. 2, Online Resource 4) make explicit, and only within-procedure, time-aware adjustment isolates a credible approach effect. To our knowledge no comparable study has applied a uniform stabilized-IPTW methodology with indication-aware cancer-site adjustment across 13 distinct oncologic procedure groups within a single dataset.

**Fig. 3:**
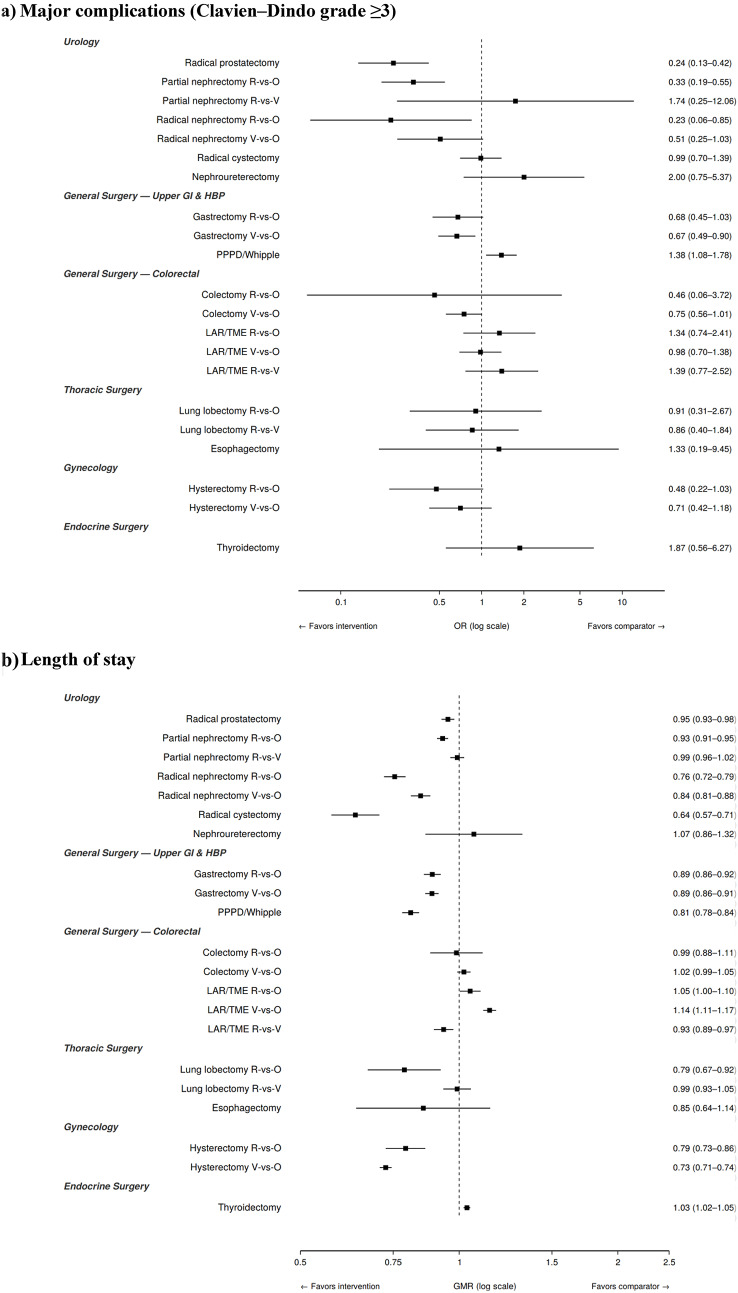
Forest plots of the two primary perioperative outcomes across 22 procedure-level contrasts adjusted by stabilized inverse probability of treatment weighting, grouped by surgical department. a major complications (Clavien–Dindo grade ≥ 3, odds ratios) and b length of stay (geometric mean ratios). Comparison codes: R robot-assisted surgery, V video-assisted surgery, O open. Squares denote point estimates, horizontal lines denote 95% confidence intervals and the dashed vertical line marks the null value. The remaining outcomes (perioperative transfusion, 30-day readmission and operation time) are shown in Online Resource 5

The most comprehensive multi-domain benefit was observed for RAS partial nephrectomy, with concurrent reductions in major complications (odds ratio 0.33, 0.19–0.55), transfusion (0.50) and readmission (0.30). Peyronnet et al. reached the same conclusion by the same analytic route: across 1,800 partial nephrectomies they reported an inverse-probability-weighted odds ratio of 2.11 (1.53–2.91) for complications after open compared with robotic surgery, which inverts to 0.47 in our direction [17]. Our estimate is the lower of the two because we counted only CDC grade ≥ 3 events whereas they counted all complications, and severe events are where the robotic advantage in this procedure appears concentrated. The contrast against VAS was less consistent: we found no difference (1.74, 0.25–12.06), whereas Khalifeh et al. reported fewer postoperative complications after robotic than laparoscopic partial nephrectomy (24.5% vs. 32.0%) [18]. That series was performed by one high-volume surgeon over a decade in which laparoscopic partial nephrectomy was being displaced, so its laparoscopic arm is unlikely to represent contemporary practice; our VAS arm, at 155 procedures, also yields intervals too wide to exclude a moderate effect.

For radical prostatectomy every measured outcome favored RAS, including major complications (0.24, 0.13–0.42), transfusion (0.09) and readmission (0.31). The only randomized comparison against open retropubic prostatectomy found the same direction but could not establish significance: Yaxley et al. observed postoperative complications in 4% versus 9% of men (*p* = 0.052) and intraoperative adverse events in 2% versus 8% among 308 operated patients [19]. With 4,386 robot-assisted and 464 open procedures we had precision that a 300-patient trial did not. Both studies also found operative time to be shorter rather than longer with the robot for this one procedure — the exception among our 13 groups — consistent with radical prostatectomy being the most mature robotic program in either setting.

For radical cystectomy our results reproduced the RAZOR trial in every domain it reported: no difference in major complications (0.99, 0.70–1.39, against adverse events in 67% versus 69% of trial patients), significantly less transfusion, significantly shorter stay and significantly longer operative time [20]. Concordance between a randomized trial and a weighted real-world cohort across all four domains is reassuring for both.

The elevated complication (1.38, 1.08–1.78) and readmission (1.63, 1.20–2.22) estimates for RAS pancreatoduodenectomy run against the general pattern, and the period-stratified analysis (Online Resource 6) localized both to the 2021–2023 program-expansion window, with resolution toward the null in 2024–2025. A minimally invasive disadvantage of similar direction and magnitude appeared in LEOPARD-2, in which CDC grade ≥ 3 complications occurred after 50% of laparoscopic versus 39% of open pancreatoduodenectomies (risk ratio 1.29, 0.82–2.02) and the trial was halted for excess complication-related mortality; its investigators likewise attributed the finding to experience, learning curve and annual volume rather than to the approach itself [21]. This is the pattern that frameworks for evaluating surgical innovation anticipate during the assessment phase [22], and ROLARR reached a parallel conclusion by a parallel method, its prespecified learning-effect analysis showing benefit concentrated among surgeons with the greatest robotic experience [23]. Time-aware sensitivity analysis is therefore not optional in observational comparisons of an approach still being adopted.

Not every signal favored the robot. For low anterior resection and total mesorectal excision the RAS-versus-VAS contrast was null to unfavorable for major complications (1.39, 0.77–2.52) and readmission (1.91, 0.99–3.71), matching ROLARR, in which the adjusted odds ratio for 30-day postoperative complications was 1.04 (0.69–1.58), conversion was not significantly reduced and length of stay was identical between arms [23]. Our operative-time penalty was far larger than theirs (geometric mean ratio 1.60 versus roughly 1.14), plausibly because ROLARR admitted only surgeons who had already performed at least ten robotic rectal resections, and whose median prior robotic experience was 50 cases, whereas our institution-wide series spans the entire adoption curve from its first case. Thyroidectomy likewise showed no complication benefit (1.87, 0.56–6.27) and a numerically higher readmission estimate, while carrying the largest operative-time penalty in the portfolio (geometric mean ratio 1.70, 1.67–1.74). Sung et al. weighted 722 total thyroidectomies at another Korean center by the same method and found that the robotic approach did not influence clinical recurrence (odds ratio 0.784, 0.150–3.403) [24]. Neither a perioperative nor an oncologic advantage is therefore apparent for this procedure, and the case for the robotic approach rests instead on avoidance of a visible cervical scar, which matters to a young, predominantly female population. For gastrectomy our RAS and VAS contrasts against open were nearly identical (0.68 and 0.67), implying no incremental benefit of the robot over laparoscopy, whereas Ojima et al. reported grade ≥ II complications after 8.8% of robotic versus 19.7% of laparoscopic gastrectomies in a two-center randomized trial [25]. Their laparoscopic complication rate is roughly twice the crude rate in our laparoscopic arm, so the divergence may lie in the comparator rather than in the robot.

The most consistent finding across the portfolio was reduced perioperative transfusion, which matters beyond the immediate perioperative period because allogeneic transfusion has itself been associated with adverse oncologic outcomes after cancer surgery [26]. Length of stay behaved differently, and one cross-cutting caution follows. Our reductions were largest where the open pathway was longest — radical cystectomy, with an open median of 21 days against 10 days for RAS — and smallest where local practice already imposed a floor, as in radical prostatectomy, where all three approaches shared a 5-day median and the geometric mean ratio was 0.95. Yaxley et al. reported 1.55 versus 3.27 days for that same procedure, a far larger proportional difference in a setting with much shorter baseline stays [19]. Length of stay is therefore bounded by local discharge convention as much as by surgical invasiveness, and proportional reductions should not be transported across health systems without that caveat.

For institutional planning the practical implication is that robotic capacity should be prioritized procedure by procedure rather than adopted or rejected wholesale. In our portfolio the most robust perioperative return was in partial nephrectomy and radical prostatectomy, whereas the gastrointestinal contrasts against laparoscopy showed little incremental benefit at a consistent operative-time cost.

### Strengths and limitations

This study has several strengths. First, the data span ten years. Second, the portfolio-level view was obtained by reproducible extraction from a single CDW. Third, the uniform pipeline allowed all 13 procedure groups to be assembled and analyzed identically, end to end. Fourth, the analytic decisions at each stage — indication-aware cancer-site adjustment, the choice of balance thresholds and the descriptive treatment of operation time — were made with input from the participating surgical specialties rather than from the warehouse data alone.

The study also has limitations. First, the cohort was assembled from the central CDW rather than from prospective specialty registries; dedicated audited registries such as established surgical quality-improvement programs achieve higher data fidelity than warehouse extraction [27]. Second, only CDC grade ≥ 3 events were captured, because grade 1–2 events resolve without further intervention and are not reliably recorded in warehouse sources, and mortality was ascertained as in-hospital death through the data-extract date of 31 December 2025 with no out-of-hospital death surveillance, so later deaths are not counted. Third, operation time is a post-treatment mediator confounded by learning curve and case complexity, and the operative-time ratios reported here should be read descriptively rather than causally. Fourth, residual confounding cannot be excluded despite stabilized IPTW, and the nine comparisons with inadequate balance yield exploratory results only; cancer-site adjustment materially affected that judgement, since for colectomy the site flags revealed a substantial case-mix imbalance in the robotic arm (74.2% right colon versus 59.3–63.3% in the open and VAS arms; Online Resource 7) and for hysterectomy they moved the comparison to an exploratory designation. Fifth, no subanalyses of demographic or other potential effect modifiers were performed. Finally, our results are not necessarily generalizable, although the process by which we arrived at them is a reproducible framework that other institutions can emulate.

### Future directions

Two related bodies of work are logical next steps. The first is to collect and compare these patients’ long-term oncologic outcomes against their short-term outcomes. The second is a patient-centerd cost-benefit analysis. Globally, patients are increasingly involved in collaborative care, which includes considering a procedure’s direct cost as well as its safety profile. Patients are interested in a procedure’s incidence of major complications, intensive care admission, readmission, transfusion and LOS, which should be integrated into a single appraisal so that the perioperative trade-offs of each approach are transparent.

In the Republic of Korea, private indemnity insurance frequently reimburses robot-assisted oncologic surgery. This has driven high patient uptake, yet a covered procedure tells a patient little about whether the price is commensurate with its perioperative safety advantages. The economic question is not hypothetical: the ROLARR health economic analysis found robotic rectal resection unlikely to be cost-saving, with a mean difference of about US$1,100 per operation driven by theater time and instrument costs [23], and operating-room time is a principal driver of procedural cost generally [28]. Pairing the procedure-level safety estimates reported here with procedure-level cost data would give objective, procedure-specific evidence to inform that choice.

## Conclusion

Across 13 oncologic procedure groups over ten years, RAS and VAS approaches were associated overall with consistent reductions in transfusion, plus procedure-specific benefits, most comprehensively in RAS partial nephrectomy and radical prostatectomy. Because aggregate technology-level comparison is confounded by procedure mix and adoption timing, procedure-level stabilized IPTW rather than technology-level pooling should guide interpretation and technology investment. Period-stratified analysis can distinguish the source of an apparent complication signal, for example by showing that it is confined to a single program-expansion phase that resolves with maturation. This underscores the importance of temporal sensitivity analysis in comparative effectiveness research.

## Supplementary Information

Below is the link to the electronic supplementary material.

Supplementary Material 1

Supplementary Material 2

Supplementary Material 3

Supplementary Material 4

Supplementary Material 5

Supplementary Material 6

Supplementary Material 7

## Data Availability

The data supporting the findings of this study were extracted from the clinical data warehouse of Seoul National University Hospital. The dataset consists of patient-level clinical records and is not publicly available, because public release would compromise patient privacy and is prohibited under institutional data-protection policy. Access is governed by the Institutional Review Board and the Data Review Board of Seoul National University Hospital; enquiries regarding the data may be directed to the corresponding author.
