# Supplementary material for: Perioperative outcomes of robot-assisted, laparoscopic or thoracoscopic, and open surgery across 13 cancer operations: a single-institution cohort of 36,208 cases
Source: J Robot Surg. 2026 Aug 17;20(1):845. doi: 10.1007/s11701-026-03814-7 (PMC13481418; doi:10.1007/s11701-026-03814-7)
Supplement: Supplementary file 1 [file 11701_2026_3814_MOESM1_ESM.pdf]

Journal of Robotic Surgery

Perioperative Outcomes of Robot-Assisted, Laparoscopic or Thoracoscopic, and Open Surgery Across 13 Cancer Operations: A Single-Institution Cohort of 36,208 Cases

Hojun Yu, Kyung Hee Han, Soyoun Kim, Chang Wook Jeong

Corresponding author: Chang Wook Jeong, Department of Urology, Seoul National University Hospital, 101 Daehak-ro, Jongno-gu, Seoul 03080, Republic of Korea. E-mail drboss@snu.ac.kr

Online Resource 1 Unadjusted baseline characteristics of the analytic cohort

Variable	Open	VAS	RAS	Total	SMD (unadjusted)[a]	P value[b]
Age, years	60.3 (13.2)	61.2 (12.1)	61.6 (12.9)	61.0 (12.7)	0.101	<0.001
Body mass index, kg/m²	24.0 (3.3)	24.1 (3.2)	24.5 (3.1)	24.2 (3.2)	0.135	<0.001
Charlson Comorbidity Index	2.3 (0.8)	2.2 (0.6)	2.1 (0.7)	2.2 (0.7)	0.236	<0.001
Pathologic T stage (numeric)[c]	2.1 (1.1)	1.9 (1.0)	1.9 (0.8)	2.0 (1.0)	0.198	<0.001
ASA physical status						<0.001
1–2	10,052 (86.8)	12,076 (89.6)	10,218 (91.6)	32,346 (89.3)		
≥3	1,506 (13.0)	1,381 (10.2)	925 (8.3)	3,812 (10.5)	0.154	
Female sex, n (%)	5,789 (50.0)	7,423 (55.1)	3,224 (28.9)	16,436 (45.4)	0.551	<0.001
Prior abdominal surgery, n (%)	163 (1.4)	180 (1.3)	122 (1.1)	465 (1.3)	0.028	0.089
Prior oncologic surgery, n (%)	731 (6.3)	382 (2.8)	336 (3.0)	1,449 (4.0)	0.167	<0.001
Calendar year of surgery[d]						<0.001
Early (2016–2020), n = 17,552	7,125 (40.6)	6,686 (38.1)	3,741 (21.3)	100.0	0.644	
Mid (2021–2023), n = 12,425	3,196 (25.7)	4,723 (38.0)	4,506 (36.3)	100.0		
Late (2024–2025), n = 6,231	1,259 (20.2)	2,066 (33.2)	2,906 (46.6)	100.0		
Perioperative outcomes (unadjusted)						
Major complication (CDC ≥3), n (%)	605 (5.2)	443 (3.3)	345 (3.1)	1,393 (3.8)	—	<0.001
Perioperative transfusion, n (%)	2,090 (18.0)	893 (6.6)	807 (7.2)	3,790 (10.5)	—	<0.001
30-day readmission, n (%)	338 (2.9)	291 (2.2)	228 (2.0)	857 (2.4)	—	<0.001
In-hospital mortality (CDC 5), n (%)	40 (0.35)	14 (0.10)	9 (0.08)	63 (0.17)	—	<0.001
Length of stay, days, median (IQR)	6 (4–10)	7 (4–9)	5 (4–8)	6 (4–9)	—	<0.001
Estimated blood loss, mL, median (IQR)	210 (100–400)	100 (50–200)	150 (100–300)	150 (70–300)	—	<0.001
Operation time, min, median (IQR)	126 (88–174)	149 (110–195)	132 (95–184)	138 (98–185)	—	<0.001
Conversion to open or thoracotomy, n (%)	—	113 (0.84)	20 (0.18)	162 (0.45)	—	<0.001

Values are mean (SD) or median (IQR) for continuous variables and n (%) for categorical variables; no weighting applied

[a] Balance was assessed by the standardized mean difference (SMD); an unweighted SMD >0.10 indicates baseline imbalance addressed by weighting

[b] P values are from one-way analysis of variance (continuous) or the chi-square test (categorical)

[c] Pathologic T stage is reported for the subset with comparable TNM staging (n = 31,306); procedures staged by other systems, such as hysterectomy, are excluded from this row

[d] Calendar year is categorical; each period row shows the within-period approach composition, so rows sum to 100%. The SMD is computed on year as a continuous covariate

ASA American Society of Anesthesiologists, CDC Clavien–Dindo classification, IQR interquartile range, RAS robot-assisted surgery, SMD standardized mean difference, VAS video-assisted surgery
