# Supplementary material for: Perioperative outcomes of robot-assisted, laparoscopic or thoracoscopic, and open surgery across 13 cancer operations: a single-institution cohort of 36,208 cases
Source: J Robot Surg. 2026 Aug 17;20(1):845. doi: 10.1007/s11701-026-03814-7 (PMC13481418; doi:10.1007/s11701-026-03814-7)

Journal of Robotic Surgery

Perioperative Outcomes of Robot-Assisted, Laparoscopic or Thoracoscopic, and Open Surgery Across 13 Cancer Operations: A Single-Institution Cohort of 36,208 Cases

Hojun Yu, Kyung Hee Han, Soyoun Kim, Chang Wook Jeong

Corresponding author: Chang Wook Jeong, Department of Urology, Seoul National University Hospital, 101 Daehak-ro, Jongno-gu, Seoul 03080, Republic of Korea. E-mail drboss@snu.ac.kr

Online Resource 2 Standardized mean differences of covariates before and after stabilized inverse probability of treatment weighting, shown as a Love plot

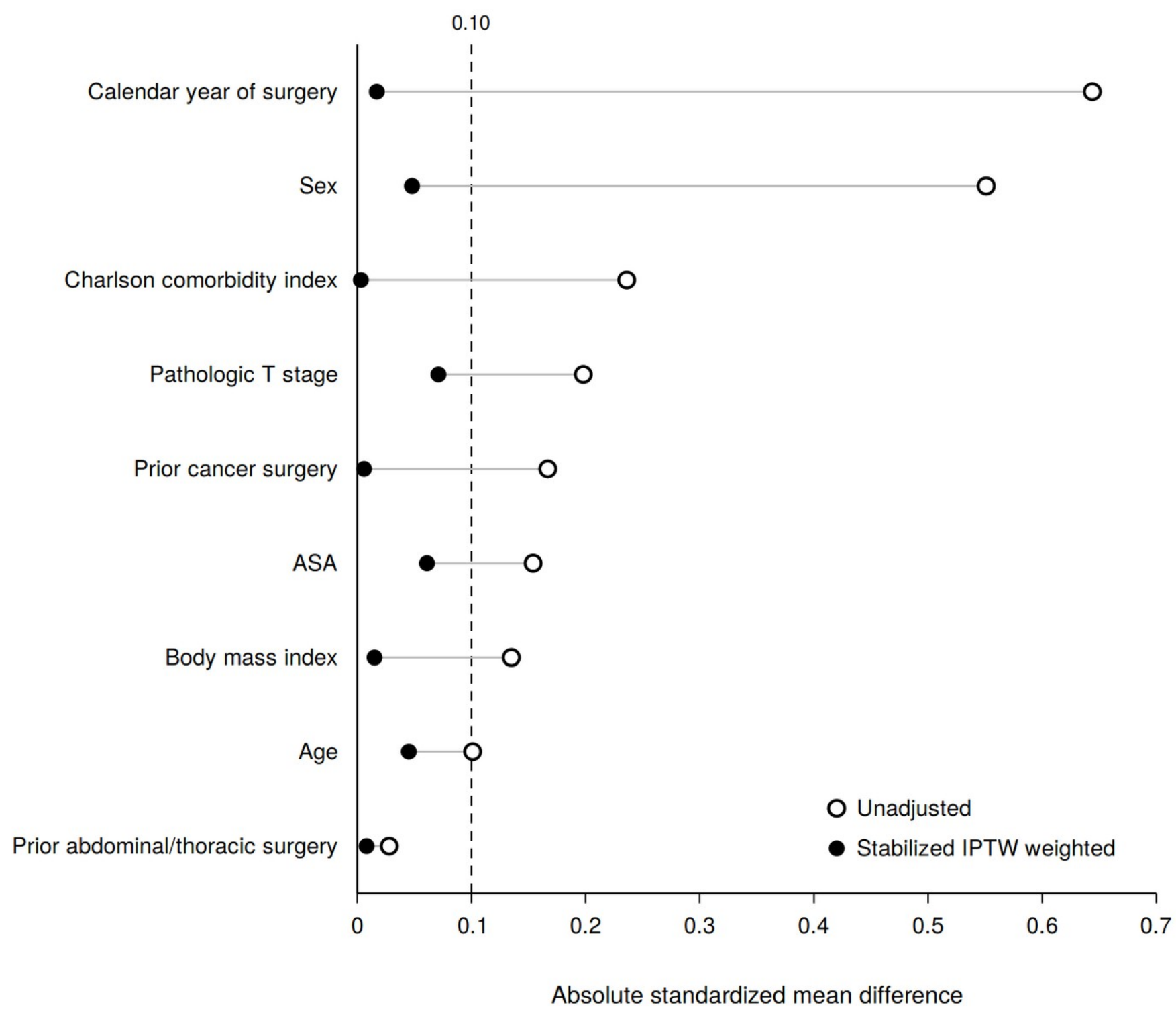

Supplement: Supplementary file 2 [file 11701_2026_3814_MOESM2_ESM.pdf]
