# Supplementary material for: Perioperative outcomes of robot-assisted, laparoscopic or thoracoscopic, and open surgery across 13 cancer operations: a single-institution cohort of 36,208 cases
Source: J Robot Surg. 2026 Aug 17;20(1):845. doi: 10.1007/s11701-026-03814-7 (PMC13481418; doi:10.1007/s11701-026-03814-7)
Supplement: Supplementary file 3 [file 11701_2026_3814_MOESM3_ESM.pdf]

Journal of Robotic Surgery

Perioperative Outcomes of Robot-Assisted, Laparoscopic or Thoracoscopic, and Open Surgery Across 13 Cancer Operations: A Single-Institution Cohort of 36,208 Cases

Hojun Yu, Kyung Hee Han, Soyoun Kim, Chang Wook Jeong
Corresponding author: Chang Wook Jeong, Department of Urology, Seoul National University Hospital, 101 Daehak-ro, Jongno-gu, Seoul 03080, Republic of Korea. E-mail drboss@snu.ac.kr

Online Resource 3 Surgical approach distribution and unadjusted perioperative outcomes by procedure group

Procedure group	Total	Open, n (%)	VAS, n (%)	RAS, n (%)	CDC ≥3 (%) O/V/R	Transfusion (%) O/V/R	LOS, median days O/V/R	30-day readmission (%) O/V/R
Radical prostatectomy	4,874	464 (9.5)	24 (0.5)	4,386 (90.0)	3.9 / 4.2 / 1.0	28.9 / 16.7 / 3.9	5 / 5 / 5	2.6 / 4.2 / 0.8
Partial nephrectomy	2,470	755 (30.6)	155 (6.3)	1,560 (63.2)	4.4 / 1.3 / 1.3	15.6 / 8.4 / 7.6	5 / 5 / 5	3.3 / 0.6 / 1.1
Radical nephrectomy	1,144	564 (49.3)	369 (32.3)	211 (18.4)	5.5 / 3.0 / 2.4	39.9 / 14.9 / 12.3	5 / 5 / 4	2.7 / 1.4 / 1.4
Radical cystectomy	1,080	404 (37.4)	1 (0.1)	675 (62.5)	15.6 / 0.0 / 12.7	65.1 / 100.0 / 27.1	21 / 16 / 10	5.7 / 0.0 / 4.9
Nephroureterectomy	437	258 (59.0)	104 (23.8)	75 (17.2)	6.2 / 2.9 / 10.7	25.2 / 18.3 / 22.7	6 / 5 / 5	3.9 / 1.9 / 6.7
Gastrectomy	6,428	1,252 (19.5)	4,133 (64.3)	1,043 (16.2)	5.2 / 3.4 / 3.1	11.8 / 4.2 / 2.3	10 / 9 / 9	2.7 / 2.2 / 2.0
Pancreatoduodenectomy	2,279	1,365 (59.9)	27 (1.2)	887 (38.9)	10.0 / 14.8 / 11.8	36.0 / 44.4 / 18.8	12 / 12 / 10	5.6 / 7.4 / 7.9
Colectomy	2,676	1,382 (51.6)	1,263 (47.2)	31 (1.2)	9.0 / 5.9 / 6.5	17.3 / 11.9 / 19.4	8 / 8 / 8	5.1 / 4.3 / 3.2
Low anterior resection / TME	2,291	1,171 (51.1)	932 (40.7)	188 (8.2)	6.9 / 6.7 / 8.0	10.9 / 6.3 / 8.0	7 / 8 / 7	4.3 / 4.2 / 6.9
Lung lobectomy and segmentectomy	3,667	251 (6.8)	3,080 (84.0)	336 (9.2)	5.2 / 2.8 / 2.1	15.9 / 2.0 / 2.7	8 / 6 / 5	2.0 / 1.7 / 1.2
Esophagectomy	296	39 (13.2)	19 (6.4)	238 (80.4)	2.6 / 0.0 / 4.2	30.8 / 10.5 / 15.1	20 / 16 / 17	8.6 / 0.0 / 7.8
Hysterectomy	4,456	842 (18.9)	3,284 (73.7)	330 (7.4)	1.8 / 1.7 / 2.4	25.1 / 10.4 / 9.7	5 / 3 / 4	1.2 / 1.3 / 1.8
Thyroidectomy	4,110	2,833 (68.9)	84 (2.0)	1,193 (29.0)	0.3 / 1.2 / 0.3	0.5 / 1.2 / 0.2	3 / 4 / 3	0.1 / 1.2 / 0.3

Values are n (%) for approach distribution and crude percentages or medians for outcomes, by approach within each procedure group. Outcome triples are ordered Open / VAS / RAS

Lung lobectomy includes segmentectomy; hysterectomy includes total abdominal hysterectomy with bilateral salpingo-oophorectomy and combined procedures

CDC Clavien–Dindo classification, LOS length of stay, O open, R robot-assisted surgery, RAS robot-assisted surgery, TME total mesorectal excision, V video-assisted surgery, VAS video-assisted surgery
