# Supplementary material for: Perioperative outcomes of robot-assisted, laparoscopic or thoracoscopic, and open surgery across 13 cancer operations: a single-institution cohort of 36,208 cases
Source: J Robot Surg. 2026 Aug 17;20(1):845. doi: 10.1007/s11701-026-03814-7 (PMC13481418; doi:10.1007/s11701-026-03814-7)
Supplement: Supplementary file 5 [file 11701_2026_3814_MOESM5_ESM.pdf]

Journal of Robotic Surgery

Perioperative Outcomes of Robot-Assisted, Laparoscopic or Thoracoscopic, and Open Surgery Across 13 Cancer Operations: A Single-Institution Cohort of 36,208 Cases

Hojun Yu, Kyung Hee Han, Soyoun Kim, Chang Wook Jeong

Corresponding author: Chang Wook Jeong, Department of Urology, Seoul National University Hospital, 101 Daehak-ro, Jongno-gu, Seoul 03080, Republic of Korea. E-mail drboss@snu.ac.kr

Online Resource 5 Forest plots of the remaining perioperative outcomes across 22 procedure-level contrasts adjusted by stabilized inverse probability of treatment weighting, grouped by surgical department. a perioperative transfusion, b 30-day readmission (odds ratios) and c operation time (geometric mean ratio). R robot-assisted surgery, V video-assisted surgery, O open

a

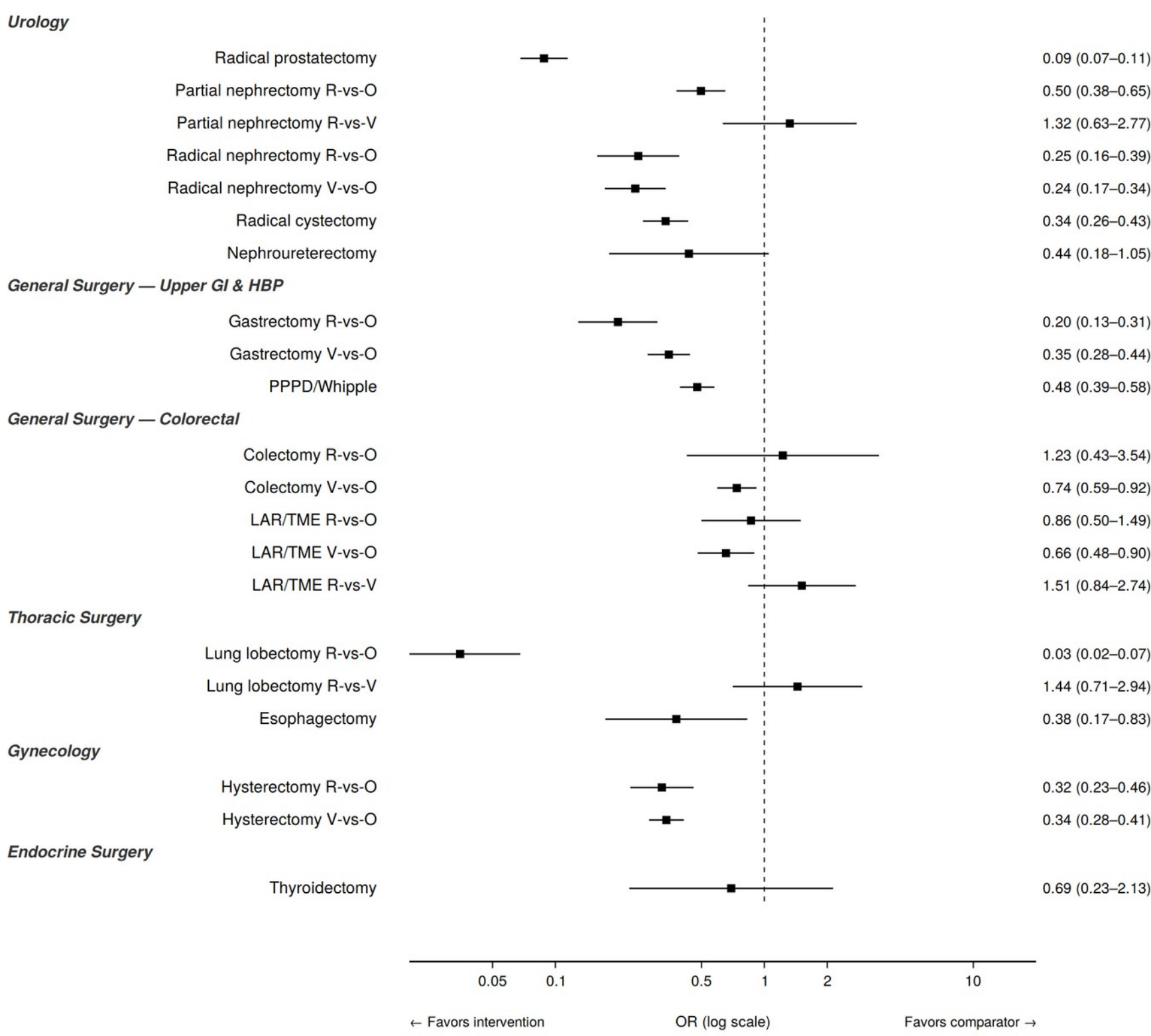

b

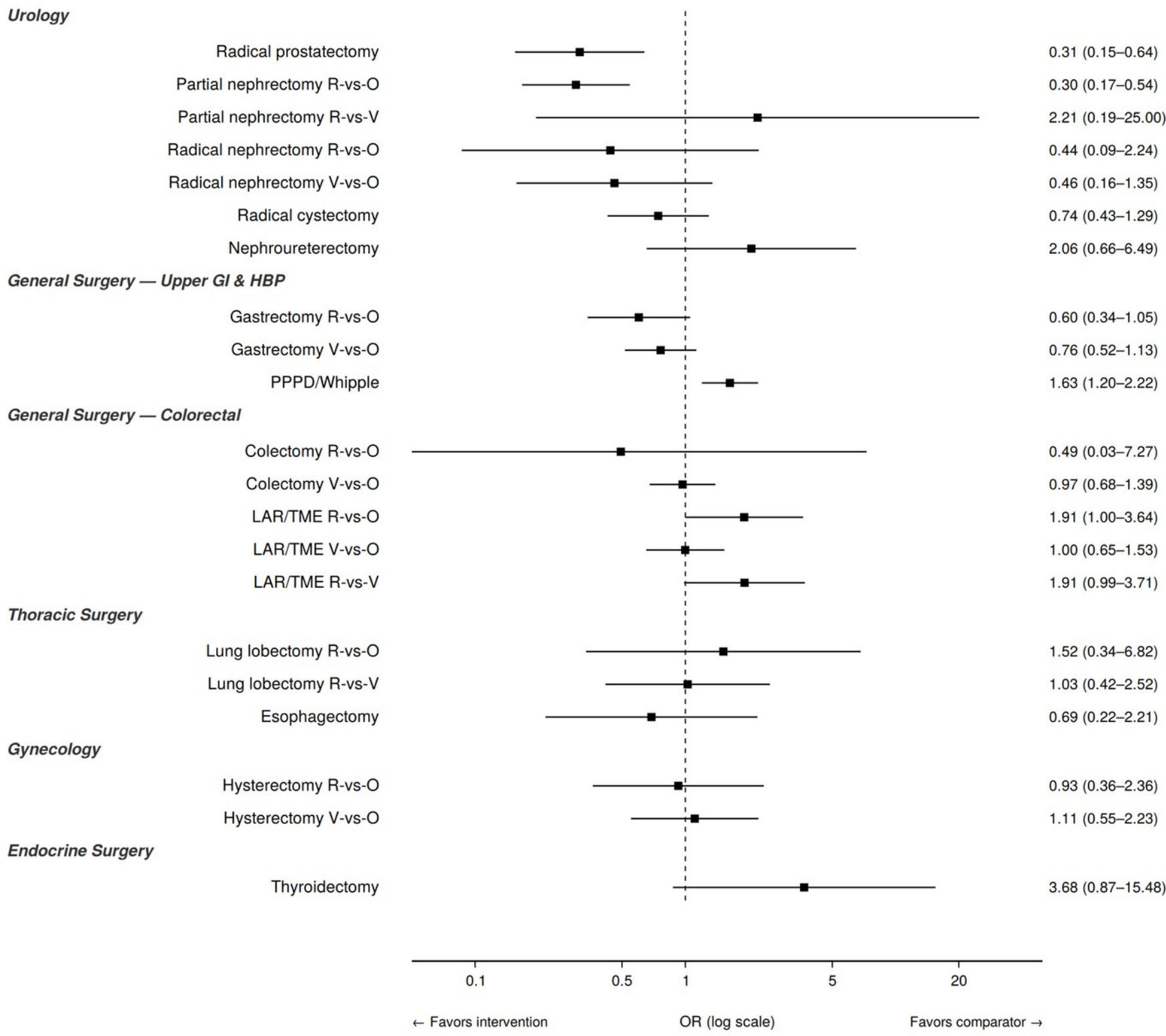
Urology
Radical prostatectomy 0.31 (0.15–0.64)
Partial nephrectomy R-vs-O 0.30 (0.17–0.54)
Partial nephrectomy R-vs-V 2.21 (0.19–25.00)
Radical nephrectomy R-vs-O 0.44 (0.09–2.24)
Radical nephrectomy V-vs-O 0.46 (0.16–1.35)
Radical cystectomy 0.74 (0.43–1.29)
Nephroureterectomy 2.06 (0.66–6.49)
General Surgery — Upper GI & HBP
Gastrectomy R-vs-O 0.60 (0.34–1.05)
Gastrectomy V-vs-O 0.76 (0.52–1.13)
PPPD/Whipple 1.63 (1.20–2.22)
General Surgery — Colorectal
Colectomy R-vs-O 0.49 (0.03–7.27)
Colectomy V-vs-O 0.97 (0.68–1.39)
LAR/TME R-vs-O 1.91 (1.00–3.64)
LAR/TME V-vs-O 1.00 (0.65–1.53)
LAR/TME R-vs-V 1.91 (0.99–3.71)
Thoracic Surgery
Lung lobectomy R-vs-O 1.52 (0.34–6.82)
Lung lobectomy R-vs-V 1.03 (0.42–2.52)
Esophagectomy 0.69 (0.22–2.21)
Gynecology
Hysterectomy R-vs-O 0.93 (0.36–2.36)
Hysterectomy V-vs-O 1.11 (0.55–2.23)
Endocrine Surgery
Thyroidectomy 3.68 (0.87–15.48)
0.1
0.5
1
5
20
← Favors intervention
OR (log scale)
Favors comparator →

c

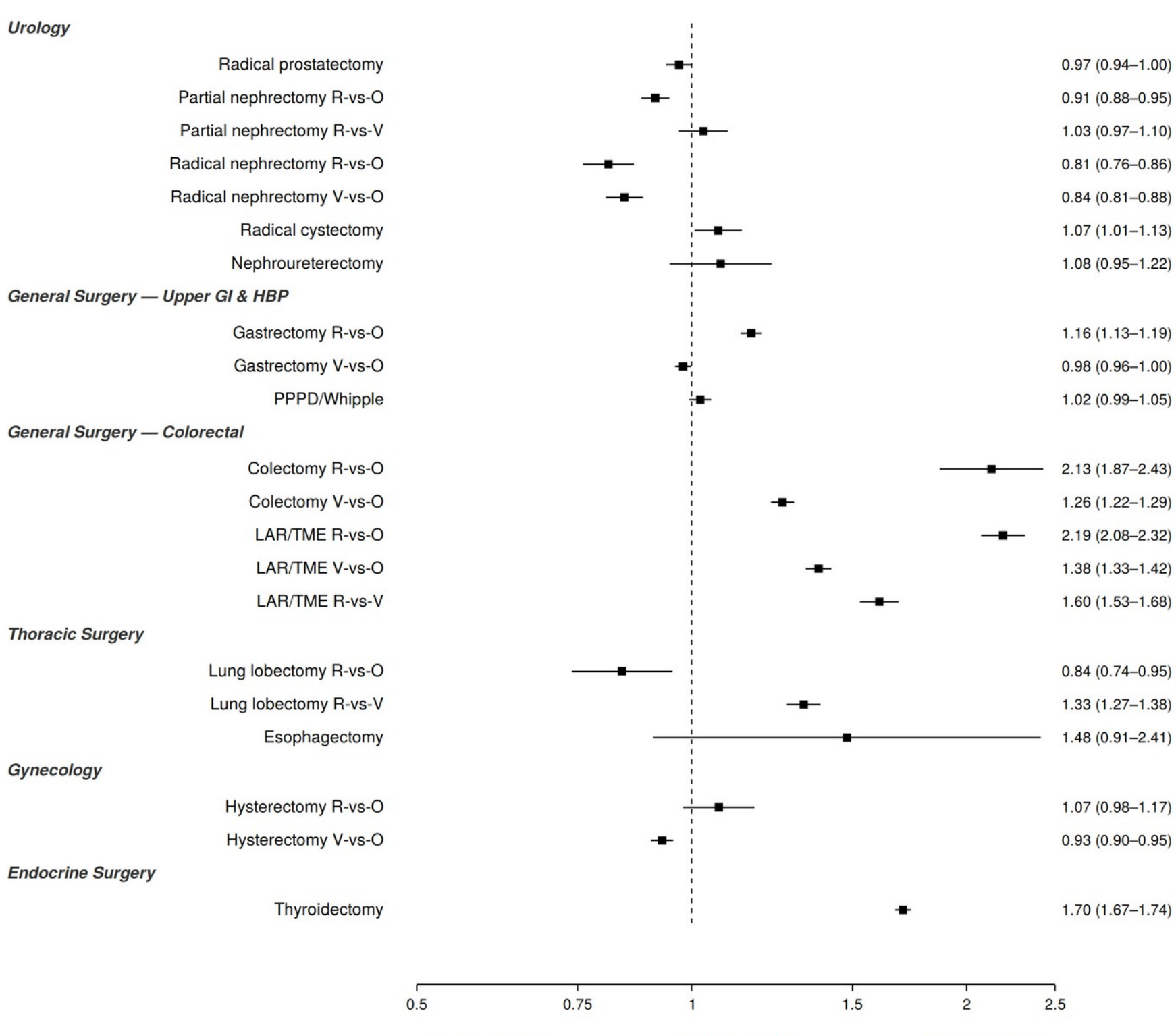
Urology
Radical prostatectomy 0.97 (0.94–1.00)
Partial nephrectomy R-vs-O 0.91 (0.88–0.95)
Partial nephrectomy R-vs-V 1.03 (0.97–1.10)
Radical nephrectomy R-vs-O 0.81 (0.76–0.86)
Radical nephrectomy V-vs-O 0.84 (0.81–0.88)
Radical cystectomy 1.07 (1.01–1.13)
Nephroureterectomy 1.08 (0.95–1.22)
General Surgery — Upper GI & HBP
Gastrectomy R-vs-O 1.16 (1.13–1.19)
Gastrectomy V-vs-O 0.98 (0.96–1.00)
PPPD/Whipple 1.02 (0.99–1.05)
General Surgery — Colorectal
Colectomy R-vs-O 2.13 (1.87–2.43)
Colectomy V-vs-O 1.26 (1.22–1.29)
LAR/TME R-vs-O 2.19 (2.08–2.32)
LAR/TME V-vs-O 1.38 (1.33–1.42)
LAR/TME R-vs-V 1.60 (1.53–1.68)
Thoracic Surgery
Lung lobectomy R-vs-O 0.84 (0.74–0.95)
Lung lobectomy R-vs-V 1.33 (1.27–1.38)
Esophagectomy 1.48 (0.91–2.41)
Gynecology
Hysterectomy R-vs-O 1.07 (0.98–1.17)
Hysterectomy V-vs-O 0.93 (0.90–0.95)
Endocrine Surgery
Thyroidectomy 1.70 (1.67–1.74)
0.5
0.75
1
1.5
2
2.5
← Favors intervention
GMR (log scale)
Favors comparator →
