# Supplementary material for: Perioperative outcomes of robot-assisted, laparoscopic or thoracoscopic, and open surgery across 13 cancer operations: a single-institution cohort of 36,208 cases
Source: J Robot Surg. 2026 Aug 17;20(1):845. doi: 10.1007/s11701-026-03814-7 (PMC13481418; doi:10.1007/s11701-026-03814-7)
Supplement: Supplementary file 6 [file 11701_2026_3814_MOESM6_ESM.pdf]

Journal of Robotic Surgery

Perioperative Outcomes of Robot-Assisted, Laparoscopic or Thoracoscopic, and Open Surgery Across 13 Cancer Operations: A Single-Institution Cohort of 36,208 Cases

Hojun Yu, Kyung Hee Han, Soyoun Kim, Chang Wook Jeong
Corresponding author: Chang Wook Jeong, Department of Urology, Seoul National University Hospital, 101 Daehak-ro, Jongno-gu, Seoul 03080, Republic of Korea. E-mail drboss@snu.ac.kr

Online Resource 6 Period-stratified pairwise comparisons

Procedure	Period	n (RAS / open)	maxSMD	CDC ≥3 OR (95% CI)	Transfusion OR (95% CI)	LOS GMR (95% CI)	30-day readmission OR (95% CI)	Conversion[a]	Operation time GMR (95% CI)[b]
Pancreatoduodenectomy	Early (2016–2020)	304 / 754	0.158	1.09 (0.71–1.66)	0.57 (0.42–0.79)	0.78 (0.75–0.82)	1.30 (0.76–2.20)	NA	1.08 (1.05–1.11)
Pancreatoduodenectomy	Mid (2021–2023)	343 / 388	0.115	1.98 (1.25–3.13)	0.29 (0.20–0.41)	0.80 (0.75–0.85)	2.09 (1.21–3.63)	NA	1.03 (1.00–1.07)
Pancreatoduodenectomy	Late (2024–2025)	240 / 223	0.070	0.82 (0.45–1.51)	0.59 (0.39–0.89)	0.92 (0.83–1.02)	0.80 (0.37–1.73)	NA	1.20 (1.12–1.30)
Pancreatoduodenectomy	Overall (2016–2025)	887 / 1,365	0.030	1.28 (0.98–1.68)	0.47 (0.39–0.58)	0.81 (0.78–0.84)	1.51 (1.08–2.10)	NA	1.14 (1.11–1.18)
Gastrectomy	Early (2016–2020)	326 / 903	0.203	0.75 (0.38–1.49)	0.20 (0.09–0.44)	0.91 (0.87–0.96)	0.73 (0.27–2.00)	NA	1.23 (1.19–1.27)
Gastrectomy	Mid (2021–2023)	458 / 276	0.075	0.96 (0.46–2.00)	0.22 (0.10–0.47)	0.90 (0.86–0.95)	0.85 (0.35–2.06)	NA	1.13 (1.07–1.18)
Gastrectomy	Late (2024–2025)	259 / 73	0.077	0.52 (0.13–2.08)	0.17 (0.07–0.44)	0.79 (0.67–0.93)	0.59 (0.12–2.94)	NA	1.14 (1.04–1.25)
Gastrectomy	Overall (2016–2025)	1,043 / 1,252	0.048	0.73 (0.48–1.11)	0.21 (0.13–0.32)	0.83 (0.81–0.86)	0.77 (0.44–1.34)	NA	1.17 (1.14–1.19)
Colectomy	Early (2016–2020)	2 / 822	NA	NA	NA	NA	NA	NA	NA
Colectomy	Mid (2021–2023)	19 / 375	0.374	0.35 (0.03–4.27)	1.11 (0.31–3.98)	1.04 (0.89–1.21)	0.70 (0.06–8.68)	NA	2.14 (1.87–2.45)
Colectomy	Late (2024–2025)	10 / 185	0.964	0.90 (0.03–27.41)	7.00 (1.03–47.50)	0.84 (0.62–1.13)	NA	NA	2.22 (1.91–2.57)
Colectomy	Overall (2016–2025)	31 / 1,382	0.478	0.57 (0.09–3.67)	1.44 (0.53–3.89)	0.97 (0.86–1.09)	0.44 (0.03–7.15)	NA	2.09 (1.87–2.34)
Low anterior resection / TME	Early (2016–2020)	38 / 726	0.339	1.66 (0.51–5.39)	1.57 (0.51–4.78)	1.07 (1.01–1.13)	2.75 (0.83–9.10)	NA	2.30 (2.13–2.49)
Low anterior resection / TME	Mid (2021–2023)	85 / 314	0.481	1.03 (0.41–2.58)	0.54 (0.21–1.37)	1.05 (0.99–1.12)	1.41 (0.50–3.98)	NA	2.11 (1.96–2.27)
Low anterior resection / TME	Late (2024–2025)	65 / 131	0.415	0.73 (0.17–3.27)	0.35 (0.13–0.91)	1.03 (0.89–1.18)	0.85 (0.15–5.00)	NA	2.25 (2.03–2.50)
Low anterior resection / TME	Overall (2016–2025)	188 / 1,171	0.394	1.03 (0.55–1.91)	0.80 (0.46–1.40)	1.02 (0.97–1.08)	1.32 (0.65–2.67)	NA	2.13 (2.03–2.23)

Stabilized inverse probability of treatment weighting, robot-assisted versus open within each period. Values are odds ratios (OR) or geometric mean ratios (GMR) with 95% confidence intervals (CI). NA denotes an estimate that could not be computed because of zero events or insufficient arm size

[a] Conversion to open is not applicable (NA) to robot-assisted versus open comparisons

[b] Operation time is a post-treatment mediator, reported descriptively only

CDC Clavien–Dindo classification, CI confidence interval, GMR geometric mean ratio, LOS length of stay, maxSMD maximum standardized mean difference, OR odds ratio, RAS robot-assisted surgery, TME total mesorectal excision
