# Supplementary material for: Perioperative outcomes of robot-assisted, laparoscopic or thoracoscopic, and open surgery across 13 cancer operations: a single-institution cohort of 36,208 cases
Source: J Robot Surg. 2026 Aug 17;20(1):845. doi: 10.1007/s11701-026-03814-7 (PMC13481418; doi:10.1007/s11701-026-03814-7)
Supplement: Supplementary file 7 [file 11701_2026_3814_MOESM7_ESM.pdf]

Journal of Robotic Surgery

Perioperative Outcomes of Robot-Assisted, Laparoscopic or Thoracoscopic, and Open Surgery Across 13 Cancer Operations: A Single-Institution Cohort of 36,208 Cases

Hojun Yu, Kyung Hee Han, Soyoun Kim, Chang Wook Jeong

Corresponding author: Chang Wook Jeong, Department of Urology, Seoul National University Hospital, 101 Daehak-ro, Jongno-gu, Seoul 03080, Republic of Korea. E-mail drboss@snu.ac.kr

Online Resource 7 Cancer-site (ICD-10) distribution by procedure group and surgical approach

Procedure group and cancer site (ICD-10)	Open, n (%)	VAS, n (%)	RAS, n (%)
Hysterectomy[a]			
Cervical (C53)	19 (2.3)	219 (6.7)	96 (29.1)
Endometrial or corpus uteri (C54–C55)	77 (9.1)	673 (20.5)	101 (30.6)
Ovarian (C56)	14 (1.7)	27 (0.8)	4 (1.2)
Other or no gynecologic primary	732 (86.9)	2,365 (72.0)	129 (39.1)
Total	842	3,284	330
Nephroureterectomy[a]			
Renal pelvis (C65)	83 (32.2)	51 (49.0)	6 (8.0)
Ureter (C66)	145 (56.2)	36 (34.6)	19 (25.3)
Bladder (C67)	20 (7.8)	7 (6.7)	40 (53.3)
Other or unspecified	10 (3.9)	10 (9.6)	10 (13.3)
Total	258	104	75
Pancreatoduodenectomy[a]			
Pancreas (C25)	712 (52.2)	11 (40.7)	316 (35.6)
Bile duct (C24, excluding ampulla)	327 (24.0)	9 (33.3)	171 (19.3)
Ampulla of Vater (C24.1)	149 (10.9)	2 (7.4)	153 (17.2)
Duodenum (C17)	50 (3.7)	2 (7.4)	38 (4.3)
Other or unspecified	127 (9.3)	3 (11.1)	209 (23.6)
Total	1,365	27	887
Colectomy[a,b]			
Right colon (C18.0–C18.4)	819 (59.3)	800 (63.3)	23 (74.2)
Left colon (C18.5–C18.7)	97 (7.0)	77 (6.1)	4 (12.9)
Other or unspecified colon	466 (33.7)	386 (30.6)	4 (12.9)
Total	1,382	1,263	31

Values are n (%) of procedures. Percentages are calculated within each procedure group and surgical approach, down each column within a group, and may not sum to 100 because of rounding; denominators are the group totals shown in the Total rows

[a] ICD-10 cancer-site flags were entered as additional propensity score covariates for these four heterogeneous-indication procedure groups

[b] Colectomy was additionally adjusted for right- versus left-sided tumor location

RAS robot-assisted surgery, VAS video-assisted surgery
